# Factors Influencing Depressive Symptoms in the Post-COVID-19 Pandemic Period

**DOI:** 10.7759/cureus.72394

**Published:** 2024-10-25

**Authors:** Hitomi Hirokawa-Ueda, Yuki Sawamura, Reiko Taketani, Yuka Tojo, Hisae Ono

**Affiliations:** 1 Department of Psychological Science, Graduate School of Humanities, Kwansei Gakuin University, Nishinomiya, JPN; 2 Department of Psychology, Faculty of Human Sciences, Konan Women’s University, Kobe, JPN

**Keywords:** covid-19, depressive symptoms, post-pandemic, public health, stress coping strategies, substance use

## Abstract

Background

Mental health issues associated with the COVID-19 pandemic have been widely reported, with various influencing factors identified. However, factors affecting mental health in the post-pandemic period remain unclear. Therefore, this study aimed to investigate the factors influencing depressive symptoms during the COVID-19 post-pandemic period.

Methods

An online cross-sectional survey using convenience sampling was conducted from December 5, 2023 to December 8, 2023. Basic demographic information and social factors were evaluated using a custom-designed questionnaire. Depressive symptoms were measured with the Japanese version of the Hospital Anxiety and Depression Scale, personality traits were measured with the Japanese version of the Ten Item Personality Inventory, and stress coping strategies were measured with the Japanese version of the Brief Coping Orientation to Problems Experienced Inventory. Logistic regression analysis was performed to examine the impact of gender, age, occupation, subjective economic status, COVID-19 history, presence of pre-pandemic depressive symptoms, personality traits, and stress coping strategies on post-pandemic depressive symptoms by calculating ORs and CIs. All statistical analyses were performed using IBM SPSS Statistics for Windows, Version 29.0 (Released 2022; IBM Corp., Armonk, NY, USA). We set a statistical significance level of 0.05 (two-tailed).

Results

Among all participants (n = 838), lower subjective economic status (OR: 2.90, 95% CI: 1.99-4.24), frequent substance use (OR: 1.70, 95% CI: 1.17-2.46), higher self-blame (OR: 2.03, 95% CI: 1.39-2.95), higher levels of active coping (OR: 0.58, 95% CI: 0.38-0.88), higher acceptance (OR: 0.61, 95% CI: 0.38-0.98), and pre-pandemic depressive symptoms (OR: 16.84, 95% CI: 11.61-24.44) were significantly associated with post-pandemic depressive symptoms.

Conclusions

This study identified pre-pandemic depressive symptoms, lower subjective economic status, higher self-blame, and frequent substance use as risk factors for post-pandemic depressive symptoms. These findings suggest the need for social support, economic assistance, and mental health education to promote constructive stress management alternatives to substance use for the prevention of depression in the context of pandemics.

## Introduction

COVID-19 was first identified in Wuhan, Hubei Province, in the People’s Republic of China in December 2019 and subsequently spread globally. On January 30, 2020, the World Health Organization declared it a public health emergency of international concern (PHEIC) [1]. The end of the PHEIC was announced on May 5, 2023, although COVID-19 remains a global threat [2]. The first case of COVID-19 in Japan was confirmed in January 2020 [3]. Following the end of the PHEIC, COVID-19 was reclassified as a less severe infectious disease on May 8, 2023, signaling the end of the pandemic from a policy perspective [4].

The COVID-19 pandemic was accompanied by a significant increase in mental health issues worldwide, with higher prevalence rates of depression and anxiety disorders [5,6]. Various biological, social, and psychological factors have been shown to influence mental health. Individuals with preexisting psychiatric disorders, women, and young people were particularly susceptible to the impact of the pandemic [5-7]. Additionally, individuals with lower educational attainment and income, those diagnosed with COVID-19, and those at high risk of infection experienced greater psychological distress [8]. COVID-19 survivors also reported experiencing mild depression in late 2023 [9].

During the COVID-19 pandemic, neuroticism was identified as a significant predictor of increased concerns related to COVID-19 and the prolonged impact of the virus, and it was shown to be a risk factor for depressive symptoms [10,11]. Openness was also recognized as a risk factor for depression, while neuroticism positively correlated with both depressive symptoms and the use of avoidant coping strategies [11,12]. Additionally, behavioral disengagement and self-blame were identified as factors that heightened the risk of developing depressive symptoms [13].

In contrast, personality traits such as extraversion, agreeableness, and conscientiousness served as protective factors against depression [11]. Extraversion was linked to seeking social support as a coping strategy, which may have helped alleviate depressive symptoms [12]. Furthermore, coping strategies such as denial, the use of instrumental support, and planning showed potential as protective factors against depressive symptoms [13].

Less is known about mental health after the COVID-19 pandemic. Therefore, this study aims to investigate factors affecting the occurrence of depressive symptoms during the post-pandemic period.

## Materials and methods

Participants

Participants were recruited for an online cross-sectional survey using non-probabilistic sampling, “A Retrospective Survey Study on the Impact of Psychological Traits on Mental Health Changes During the COVID-19 Pandemic” (UMIN000053002), via a crowdsourcing service (Lancers: www.lancers.jp/help/terms) from December 5, 2023 to December 8, 2023. In this study, 1,000 participants were planned based on feasibility. They were recruited from all over Japan, had financial accounts in Japan, and were fully proficient in Japanese. The inclusion criteria for this study were that participants needed to be 18 years of age or older and to provide informed consent after understanding the purpose of the study. The following participants were excluded from the analysis: participants who responded “Other” or “Prefer not to answer” for gender, “Other” for occupation, “Don’t know” for COVID-19 status, or provided incomplete responses to assessment scales.

Survey procedures

Participants were recruited through Lancers, where they were provided with a link to an online questionnaire created using Qualtrics (www.qualtrics.com/jp). Through the Qualtrics platform, participants were informed of the study’s purpose and gave their consent before engaging with the questionnaire. Those who completed the survey received a reward of 200 Japanese yen. This study was approved by the Kwansei Gakuin University Institutional Review Board for Medical and Biological Research Involving Human Subjects (approval number KG-IRB-23-04) on November 22, 2023, and was conducted in accordance with the principles outlined in the Declaration of Helsinki.

Measures

Data on gender, age, occupation, subjective economic status, and COVID-19 history were collected. Gender options included “Male,” “Female,” “Other,” and “Prefer not to answer.” Age was categorized into “Young adults” (18-34 years), “Middle-aged adults” (35-64 years), and “Older adults” (65 years and older). The occupation was categorized into 19 options: the 15 types classified by the Ministry of Health, Labour and Welfare [14], as well as “Student,” “Homemaker,” “Unemployed,” and “Other.” Respondents were classified as “employed” if they selected any of the 15 job types and “unemployed” if they selected “Student,” “Homemaker,” or “Unemployed.” Participants were provided with five options for economic status: “Very comfortable,” “Somewhat comfortable,” “Average,” “Somewhat difficult,” and “Very difficult.” Responses of “Very comfortable,” “Somewhat comfortable,” and “Average” were classified as “Above average,” and those of “Somewhat difficult” and “Very difficult” were classified as “Below average.” COVID-19 history responses were “Yes,” “No,” and “Don’t know.”

Personality traits were assessed using the Japanese version of the Ten Item Personality Inventory (TIPI-J). The TIPI-J is a validated self-report scale consisting of 10 pairs of traits used to assess the big five personality dimensions (extraversion, agreeableness, conscientiousness, neuroticism, and openness) [15,16]. Each pair of traits was rated on a 7-point Likert scale from “Strongly disagree (1)” to “Strongly agree (7)” [15,16]. Participants were categorized into high and low groups for each trait in accordance with previous research [16]. The permission for use can be obtained by referencing the specified papers [15,16].

Stress coping strategies were assessed using the Japanese version of the Brief Coping Orientation to Problems Experienced (Brief COPE) Inventory, a validated self-report scale consisting of 28 items in 14 subscales (Self-Distraction, Active Coping, Denial, Substance Use, Using Emotional Support, Using Instrumental Support, Behavioral Disengagement, Venting, Positive Reframing, Planning, Humor, Acceptance, Religion, and Self-Blame) [17,18]. Each item was rated on a 4-point Likert scale from “I usually don’t do this at all (1)” to “I usually do this a lot (4)” [17,18]. Participants were categorized into high- and low-frequency groups for each subscale in accordance with previous research [18]. Permission for use can be obtained by referencing the specified papers [17,18].

Depressive symptoms were assessed using the seven items related to depression from the Japanese version of the Hospital Anxiety and Depression Scale (HADS-D) [19-21]. HADS-D is a validated self-report scale, with each item rated on a 4-point Likert scale (0-3 points) and higher total scores indicating more severe depressive symptoms [19-21]. Participants with total scores of less than 8 were classified as having no depressive symptoms, and those with total scores of 8 or higher were classified as having depressive symptoms [19]. Permission for use can be obtained by referencing the specified papers [19-21]. In addition to completing the scale for the present period (December 2023), participants were asked to recall the pre-pandemic period (before November 2019) and respond based on their typical state at that time. These responses were used to determine whether participants experienced depressive symptoms in the pre-pandemic period, using the same cutoff score of 8 points.

Outcomes

The primary outcome was the influence of gender, age, occupation, subjective economic status, COVID-19 history, presence of pre-pandemic depressive symptoms, expression of different personality traits, and use of stress coping strategies on the presence of depressive symptoms in the COVID-19 post-pandemic period. The secondary outcomes were the influence of the same factors on the pre-pandemic depressive group and the pre-pandemic non-depressive group.

Statistical analysis

Logistic regression analysis using a stepwise method (the Wald test) was performed, using the presence or absence of depressive symptoms during the post-pandemic period as the dependent variable. Independent variables included gender (male or female), age (young, middle-aged, or older adults), occupation (employed or unemployed), subjective economic status (above or below average), COVID-19 history (present or absent), pre-pandemic symptoms (depressive or non-depressive), each personality trait from the TIPI-J (high or low), and each stress coping strategy from the Brief COPE Inventory (high or low). Data are expressed as means ± standard deviations or as ORs with CIs. Data analyses were performed using IBM SPSS Statistics for Windows, Version 29.0 (Released 2022; IBM Corp., Armonk, NY, USA). Statistical significance was set at p < 0.05.

## Results

Participant characteristics

Table 1 summarizes the characteristics of the study participants. Of 1,007 participants recruited, 838 were included in the final analysis, with a mean age of 42.87 ± 10.41 years. The prevalence of depressive symptoms was 458 participants (54.65%) during the pre-pandemic period and 448 participants (53.46%) during the post-pandemic period. The total HADS-D score did not differ significantly between the pre- and post-pandemic periods, at 8.66 ± 4.14 and 8.60 ± 4.49, respectively (p = 0.074).

**Table 1:** Characteristics of the participants Age groups: young adults (18-34 years), middle-aged adults (35-64 years), and elderly (65 years and older) Brief COPE: the Japanese version of Brief Coping Orientation to Problems Experienced Inventory; HADS-D: the seven items related to depression from the Japanese version of Hospital Anxiety and Depression Scale; TIPI-J: the Japanese version of the Ten Item Personality Inventory

Characteristics	All participants (n = 838)	Pre-pandemic depressive group (n = 458)	Pre-pandemic non-depressive group (n = 380)
Gender			
Male (%)	447 (53.34)	258 (56.33)	189 (49.74)
Female (%)	391 (46.66)	200 (43.67)	191 (50.26)
Age			
Mean ± SD	42.87 ± 10.41	43.08 ± 10.51	42.62 ± 10.30
Young adults (%)	187 (22.32)	98 (21.40)	89 (23.42)
Middle-aged adults (%)	629 (75.06)	346 (75.55)	283 (74.47)
Older adults (%)	22 (2.63)	14 (3.06)	8 (2.11)
Occupation			
Employed (%)	629 (74.46)	336 (73.36)	288 (75.79)
Unemployed (%)	214 (25.54)	122 (26.64)	92 (24.21)
Subjective economic status			
Above average (%)	466 (55.61)	209 (45.63)	257 (67.63)
Below average (%)	372 (44.39)	249 (54.37)	123 (32.37)
COVID-19 infection history			
Yes (%)	290 (34.61)	150 (32.75)	140 (36.84)
No (%)	548 (65.39)	308 (67.25)	240 (63.16)
TIPI-J			
Extraversion			
Mean ± SD	6.66 ± 2.82	6.10 ± 2.65	7.32 ± 2.87
High extraversion (%)	295 (35.20)	122 (26.64)	173 (45.53)
Low extraversion (%)	543(64.80)	366 (73.36)	207 (54.47)
Agreeableness			
Mean ± SD	9.90 ± 2.28	9.46 ± 2.39	10.43 ± 2.01
High agreeableness (%)	528 (63.01)	255 (55.68)	273 (71.84)
Low agreeableness (%)	310 (36.99)	203 (44.32)	107 (28.16)
Conscientiousness			
Mean ± SD	7.67 ± 2.79	7.17 ± 2.75	8.26 ± 2.73
High conscientiousness (%)	540 (64.44)	261(56.99)	279 (73.42)
Low conscientiousness (%)	298 (35.56)	197 (43.01)	101 (26.58)
Neuroticism			
Mean ± SD	8.72 ± 2.69	9.38 ± 2.46	7.92 ± 2.73
High neuroticism (%)	336 (40.10)	218 (47.60)	118 (31.05)
Low neuroticism (%)	502 (59.90)	240 (52.40)	262 (68.95)
Openness			
Mean ± SD	7.65 ± 2.72	7.32 ± 2.63	8.05 ± 2.77
High openness (%)	319 (38.07)	151 (32.97)	168 (44.21)
Low openness (%)	519 (61.93)	307 (67.03)	212 (55.79)
Brief COPE			
Self-distraction			
Mean ± SD	5.28 ± 1.21	5.23 ± 1.16	5.33 ± 1.26
High self-distraction (%)	619 (73.87)	341 (74.45)	278 (73.16)
Low self-distraction (%)	219 (26.13)	117 (25.55)	102 (26.84)
Active coping			
Mean ± SD	5.83 ± 1.03	5.64 ± 1.02	6.07 ± 0.99
High active coping (%)	584 (69.69)	289 (63.10)	295 (77.63)
Low active coping (%)	254 (30.31)	169 (36.90)	85 (22.37)
Denial			
Mean ± SD	3.17 ± 1.17	3.34 ± 1.27	2.97 ± 1.02
High denial (%)	515 (61.46)	297 (64.85)	218 (57.37)
Low denial (%)	323 (38.54)	161 (35.15)	162 (42.63)
Substance use			
Mean ± SD	3.43 ± 1.67	3.52 ± 1.70	3.32 ± 1.64
High substance use (%)	370 (44.15)	222 (48.47)	148 (38.95)
Low substance use (%)	468 (55.85)	236 (51.53)	232 (61.05)
Using emotional support			
Mean ± SD	4.71 ± 1.46	4.53 ± 1.45	4.92 ± 1.44
High using emotional support (%)	427 (50.95)	211 (46.07)	216 (56.84)
Low using emotional support (%)	411 (49.05)	247 (53.93)	164 (43.16)
Using instrumental support			
Mean ± SD	4.89 ± 1.47	4.73 ± 1.50	5.08 ± 1.42
High using instrumental support (%)	453 (54.06)	222 (48.47)	231 (60.79)
Low using instrumental support (%)	385 (45.94)	236 (51.53)	149 (39.21)
Behavioral disengagement			
Mean ± SD	4.28 ± 1.23	4.53 ± 1.22	3.97 ± 1.16
High behavioral disengagement (%)	682 (81.38)	399 (87.12)	283 (74.47)
Low behavioral disengagement (%)	156 (18.62)	59 (12.88)	97 (25.53)
Venting			
Mean ± SD	4.68 ± 1.36	4.70 ± 1.38	4.66 ± 1.34
High venting (%)	430 (51.31)	237 (51.75)	193 (50.79)
Low venting (%)	408 (48.69)	221 (48.25)	187 (49.21)
Positive reframing			
Mean ± SD	5.27 ± 1.24	5.13 ± 1.22	5.45 ± 1.25
High positive reframing (%)	434 (51.79)	214 (46.72)	220 (57.89)
Low positive reframing (%)	404 (48.21)	244 (53.28)	160 (42.11)
Planning			
Mean ± SD	6.07 ± 1.19	5.87 ± 1.22	6.31 ± 1.12
High planning (%)	635 (75.78)	318 (69.43)	317 (83.42)
Low planning (%)	203 (24.22)	140 (30.57)	63 (16.58)
Humor			
Mean ± SD	3.91 ± 1.46	3.85 ± 1.47	3.98 ± 1.45
High humor (%)	513 (61.22)	270 (58.95)	243 (63.95)
Low humor (%)	325 (38.78)	188 (41.05)	137 (36.05)
Acceptance			
Mean ± SD	6.04 ± 1.04	5.89 ± 1.05	6.22 ± 1.00
High acceptance (%)	658 (78.52)	341 (74.45)	317 (83.42)
Low acceptance (%)	180 (21.48)	117 (25.55)	63 (16.58)
Religion			
Mean ± SD	3.38 ± 1.37	3.49 ± 1.41	3.24 ± 1.30
High religion (%)	548 (65.39)	318 (69.43)	230 (60.53)
Low religion (%)	290 (34.61)	140 (30.57)	150 (39.47)
Self-blame			
Mean ± SD	5.01 ± 1.50	5.33 ± 1.48	4.63 ± 1.44
High self-blame (%)	467 (55.73)	298 (65.07)	169 (44.47)
Low self-blame (%)	371 (44.27)	160 (34.93)	211 (55.53)
Pre-pandemic HADS-D			
Mean ± SD	8.66 ± 4.14	11.69 ± 3.09	5.01 ± 1.35
Depressive (%)	458 (54.65)	-	-
Non-depressive (%)	380 (45.35)	-	-
Post-pandemic HADS-D			
Mean ± SD	8.60 ± 4.49	11.10 ± 4.09	5.60 ± 2.81
Depressive (%)	448 (53.46)	376 (82.10)	72 (18.95)
Non-depressive (%)	390 (46.54)	82 (17.90)	308 (81.05)

Pre-pandemic depressive symptoms were experienced by 458 participants, with a mean age of 43.08 ± 10.51 years. In this group, the total HADS-D score during the pre-pandemic period was significantly higher than that in the post-pandemic period, with mean scores of 11.69 ± 3.09 and 11.10 ± 4.09, respectively (p < 0.001), although both scores were 8 or higher. The remaining 380 participants, with a mean age of 42.62 ± 10.30 years, did not experience depressive symptoms in the pre-pandemic period. In this group, the total HADS-D score during the pre-pandemic period was significantly lower than that in the post-pandemic period, with mean scores of 5.01 ± 1.35 and 5.60 ± 2.81, respectively (p = 0.002), although both scores were less than 8.

Factors influencing post-pandemic depressive symptoms among all participants

Table 2 presents the results of the logistic regression analysis. The analysis revealed that participants reporting a below-average economic status (OR: 2.90, 95% CI: 1.99-4.24, p < 0.001), high substance use (OR: 1.70, 95% CI:1.17-2.46, p < 0.005), and high self-blame (OR: 2.03, 95% CI: 1.39-2.95, p < 0.001) were significantly more likely to experience depressive symptoms in the post-pandemic period than those with an above average economic status, low substance use, and low self-blame, respectively. Participants with high active coping (OR: 0.58, 95% CI: 0.38-0.88, p = 0.011) and those with high acceptance (OR: 0.61, 95% CI: 0.38-0.98, p = 0.041) were significantly less likely to experience depressive symptoms than those with low active coping and low acceptance, respectively. The most significant influencing factor was the presence of pre-pandemic depressive symptoms (OR: 16.84, 95% CI: 11.61-24.44, p < 0.001). Personality traits had no significant impact on depressive symptoms in the post-pandemic period.

**Table 2:** Factors influencing post-pandemic depressive symptoms among all participants Wald: Wald statistic; model fit: χ² = p < 0.001; Hosmer-Lemeshow test: p = 0.214; R² = 0.541 B: regression coefficient; Brief COPE: the Japanese version of Brief Coping Orientation to Problems Experienced Inventory; HADS-D: the seven items related to depression from the Japanese version of Hospital Anxiety and Depression Scale

Predictor	B	SE	Wald	OR	95% CI		p-value
					Lower	Upper	
Subjective economic status							
Above average				Reference			
Below average	1.07	0.19	30.47	2.90	1.99	4.24	<0.001
Brief COPE							
Low active coping				Reference			
High active coping	-0.55	0.22	6.48	0.58	0.38	0.88	0.011
Low substance use				Reference			
High substance use	0.53	0.19	7.78	1.70	1.17	2.46	<0.005
Low acceptance				Reference			
High acceptance	-0.50	0.25	4.17	0.61	0.38	0.98	0.041
Low self-blame				Reference			
High self-blame	0.71	0.19	13.70	2.03	1.39	2.95	<0.001
HADS-D							
Pre-pandemic non-depressive group				Reference			
Pre-pandemic depressive group	2.82	0.19	221.07	16.84	11.61	24.44	<0.001

Factors influencing post-pandemic depressive symptoms in the pre-pandemic depressive group

Table 3 shows the results of logistic regression analysis of the impact of the independent variables on post-pandemic depressive symptoms within the pre-pandemic depressive group. The results indicated that male participants (OR: 1.76, 95% CI: 1.06-2.90, p = 0.028) and those reporting a below-average subjective economic status (OR: 1.96, 95% CI: 1.17-3.27, p = 0.011) were more likely to experience depressive symptoms in the post-pandemic period than female participants and those with an above average economic status, respectively. Participants with the personality trait of high openness (OR: 0.54, 95% CI: 0.33-0.91, p = 0.021) and those with high active coping (OR: 0.55, 95% CI: 0.31-1.00, p = 0.049) were less likely to experience depressive symptoms in the post-pandemic period than those with low openness and low active coping, respectively.

**Table 3:** Factors influencing post-pandemic depressive symptoms in the pre-pandemic depressive group Wald: Wald statistic; model fit: χ² = p < 0.001; Hosmer-Lemeshow test: p = 0.222; R² = 0.123 B: regression coefficient; Brief COPE: the Japanese version of Brief Coping Orientation to Problems Experienced Inventory; TIPI-J: the Japanese version of the Ten Item Personality Inventory

Predictor	B	SE	Wald	OR	95% CI		p-value
					Lower	Upper	
Gender							
Female				Reference			
Male	0.56	0.26	4.80	1.76	1.06	2.90	0.028
Subjective economic status							
Above average				Reference			
Below average	0.67	0.26	6.50	1.96	1.17	3.27	0.011
TIPI-J							
Low active openness				Reference			
High active openness	-0.61	0.26	5.31	0.54	0.33	0.91	0.021
Brief COPE							
Low active coping				Reference			
High active coping	-0.60	0.30	3.88	0.55	0.31	1.00	0.049

Factors influencing post-pandemic depressive symptoms in the pre-pandemic non-depressive group

Table 4 presents the results of a similar logistic regression analysis of the pre-pandemic non-depressive group. The results indicated that participants reporting a below-average economic status (OR: 4.88, 95% CI: 2.66-8.95, p < 0.001), those who were unemployed (OR: 2.40, 95% CI: 1.27-4.56, p = 0.007), those with high substance use (OR: 3.37, 95% CI: 1.85-6.14, p < 0.001), and those with high self-blame (OR: 2.58, 95% CI: 1.41-4.73, p = 0.002) were more likely to experience depressive symptoms than those with an above average economic status, those who were employed, those with low substance use, and those with low self-blame, respectively. Participants with the personality trait of high conscientiousness (OR: 0.50, 95% CI: 0.27-0.91, p = 0.024) and those with high acceptance (OR: 0.48, 95% CI: 0.24-0.96, p = 0.038) were less likely to experience depressive symptoms than those with low conscientiousness and low acceptance, respectively.

**Table 4:** Factors influencing post-pandemic depressive symptoms in the pre-pandemic non-depressive group Wald: Wald statistic; model fit: χ²= p < 0.001; Hosmer-Lemeshow test: p = 0.526; R² = 0.286 B: regression coefficient; Brief COPE: the Japanese version of Brief Coping Orientation to Problems Experienced Inventory; TIPI-J: the Japanese version of the Ten Item Personality Inventory

Predictor	B	SE	Wald	OR	95% CI		p-value
					Lower	Upper	
Subjective economic status							
Above average				Reference			
Below average	1.59	0.31	26.27	4.88	2.66	8.95	<0.001
Occupation							
Employed				Reference			
Unemployed	0.88	0.33	7.21	2.40	1.27	4.56	0.007
TIPI-J							
Low active conscientiousness				Reference			
High active conscientiousness	-0.70	0.31	5.11	0.50	0.27	0.91	0.024
Brief COPE							
Low substance use				Reference			
High substance use	1.21	0.31	15.67	3.37	1.85	6.14	<0.001
Low acceptance				Reference			
High acceptance	-0.73	0.35	4.31	0.48	0.24	0.96	0.038
Low self-blame				Reference			
High self-blame	0.95	0.31	9.48	2.58	1.41	4.73	0.002

## Discussion

This study investigated factors influencing depressive symptoms during the COVID-19 post-pandemic period. The results indicated that pre-pandemic depressive symptoms were a risk factor for post-pandemic depressive symptoms. A below-average economic status, frequent use of alcohol or drugs during times of stress, and high levels of self-blame were additional risk factors. For individuals with pre-pandemic depressive symptoms, the risk factors were male gender and a below-average economic status; for those without pre-pandemic depressive symptoms, the risk factors were a below-average economic status, unemployment, frequent use of alcohol or drugs, and high levels of self-blame.

The prevalence of depressive symptoms was 53-55%, with no significant difference between the pre- and post-pandemic periods. The high prevalence of depressive symptoms in this study may be because participants were recruited online using the survey title “A Retrospective Survey Study on the Impact of Psychological Traits on Mental Health Changes During the COVID-19 Pandemic,” attracting individuals already aware of their own mental health issues.

Participants who reported pre-pandemic depressive symptoms were approximately 17 times more likely to experience post-pandemic depressive symptoms than those who did not. Consistent with this finding, previous studies have identified psychiatric disorders as a risk factor for worsening mental health during the COVID-19 pandemic [6,7].

Among participants with pre-pandemic depressive symptoms, 82% also experienced symptoms in the post-pandemic period. Considering that approximately 50% of cases of untreated depression resolve naturally within a year [22], the persistence of depressive symptoms for approximately four years may be due to the effects of the pandemic. Additionally, being male and reporting a below-average economic status increased the risk of post-pandemic depressive symptoms by approximately 1.8 and 2 times, respectively. This suggests that social and economic support is needed for individuals experiencing depressive symptoms, as well as medical interventions.

Among participants without pre-pandemic depressive symptoms, 19% developed post-pandemic depressive symptoms. As the annual incidence rate of depression is 3-5% [23], this finding suggests that the COVID-19 pandemic may have increased the incidence of depression. However, the incidence in our study may have been affected by our self-selecting study population, who responded to a survey on COVID-19 and mental health. Having a below-average economic status, being unemployed, using alcohol or drugs frequently, and engaging in self-blame during stress increased the risk of post-pandemic depressive symptoms 4.9, 2.4, 3.4, and 2.6 times, respectively. This indicates the need for economic support and education to promote constructive stress management alternatives to substance use.

Factors that protect against post-pandemic depressive symptoms were also identified in this study. Among all participants, a higher ability to accept reality under stress was associated with a 0.6 times lower risk of depressive symptoms. Among participants with pre-pandemic depressive symptoms, high openness to new experiences and proactive stress-coping attitudes reduced the risk by 0.6 times. Among those without pre-pandemic depressive symptoms, conscientiousness and a flexible acceptance of stress were associated with a 0.5 times lower risk. These findings suggest that clinical psychological interventions focusing on personality traits and stress-coping strategies may be effective measures for the prevention of post-pandemic depressive symptoms.

This study has several limitations. First, due to its cross-sectional design, causal relationships between factors cannot be established. Additionally, the retrospective self-reporting of pre-pandemic depressive symptoms may introduce recall bias. Second, depressive symptoms were self-reported without expert assessment of symptom severity. Third, the online administration of the survey may have introduced selection bias, limiting the representativeness of the sample. Additionally, since a reward was provided for participation, there might be a possibility that social desirability bias may have arisen. Despite its limitations, this study identifies pre-pandemic depressive symptoms as the most significant risk factor for depressive symptoms following the pandemic. It is recommended that healthcare providers address self-blame and substance use through targeted stress management strategies, while policymakers should prioritize mental health support for individuals with pre-pandemic depressive symptoms and provide economic assistance. Future research should include longitudinal studies incorporating objective assessments of depressive symptoms conducted by professionals.

## Conclusions

This study empirically examined the factors influencing the incidence of post-pandemic depressive symptoms. The findings identified pre-pandemic depressive symptoms as the most significant risk factor. Among participants with pre-pandemic depressive symptoms, male gender, and a below-average economic status were identified as risk factors. For those without pre-pandemic depressive symptoms, a below-average economic status, unemployment, frequent alcohol or drug use, and self-blaming coping strategies were identified as risk factors. These findings suggest the need for social support, economic assistance, and mental health education to promote constructive stress management alternatives to substance use for the prevention of depression in the context of pandemics. This study therefore provides valuable insights for the prevention of depressive symptoms following pandemics.
